# Increasing access to psychological therapy on acute mental health wards: staff and patient experiences of a stepped psychological intervention

**DOI:** 10.1186/s12888-025-06721-7

**Published:** 2025-03-28

**Authors:** Isobel Johnston, Dawn Edge, Paul Wilson, Adele Beinaraviciute, Sandra Bucci, Richard Drake, Gill Gilworth, Gillian Haddock, Fritz Handerer, Sonalia Kaur, Karina Lovell, Helen Morley, Owen Price, Mica Samji, Katherine Berry

**Affiliations:** 1 Complex Trauma and Resilience Research Unit, Manchester Royal Infirmary, Manchester Greater Manchester Mental Health NHS Foundation Trust, Rawnsley Building, Manchester, M13 9WL UK; 2 Division of Psychology and Mental Health, University of Manchester, Coupland 1 Building, Manchester, M13 9PL UK; 3 Division of Population Health, Health Services Research and Primary Care, School of Health Sciences, University of Manchester, Williamson Building, Manchester, M13 9PL UK; 4 Division of Nursing, Midwifery and Social Work, University of Manchester, Jean McFarlane Building, Manchester, M13 9PL UK

**Keywords:** Clinical psychology, Mental health nursing, Mental health services, Interviews, Qualitative research

## Abstract

**Background:**

Psychological therapies are recommended for people with serious mental health problems. However, access is limited, particularly in inpatient mental health settings. The Talk, Understand and Listen in InPatient Settings (TULIPS) study is a large multi-centre cluster-randomised controlled trial which aimed to evaluate a stepped psychological intervention model to increase access to therapies for inpatients. This paper presents findings from the embedded process evaluation focusing on the contextual factors influencing intervention delivery.

**Methods:**

Thirty-two staff and 31 patients from wards receiving the intervention participated in semi-structured interviews. Data was analysed using reflexive thematic analysis. Staff and patient data were analysed separately but perspectives were compared and interwoven resulting in five themes.

**Results:**

Patients reported sessions with psychologists facilitated greater self-understanding and coping. Staff and patients reflected that formulations improved staff understanding of patient presentations. This understanding was associated with improved staff-patient relationships, more person focused practice and reduced conflict and burnout. Psychologists’ frequent physical presence on the wards and support of nursing teams enabled staff buy-in. However, significant barriers in resource, skill and confidence inhibited the delivery of nurse-led interventions within the stepped care model, as did perceptions that intervention delivery was outside the remit of nursing staff’s role.

**Conclusions:**

This study provides evidence that a stepped psychological intervention on acute mental health wards could improve patient coping and ward experience for patients and staff. Future studies should target nursing staff confidence and skill in the delivery of psychological interventions.

**Trial registration:**

ClinicalTrials.gov Identifier: NCT03950388. Registered 15th May 2019. https://clinicaltrials.gov/ct2/show/NCT03950388.

**Supplementary Information:**

The online version contains supplementary material available at 10.1186/s12888-025-06721-7.

## Background

Individuals with serious mental health problems should have access to evidence-based psychological therapies across the care pathway [1, 2]. Despite clinical guidelines, there is limited access to psychological therapies for these individuals within the United Kingdom, particularly within acute inpatient settings [3, 4]. The delivery of psychological therapy on acute wards presents a unique set of challenges including short stays, complex patient needs, ineffective team working and limited suitable space for conducting therapy [5, 6]. There is also no evidence-based approach for the delivery of therapy on acute mental health wards and limited research into how to overcome the aforementioned challenges.

The TULIPS (Talk Understand and Listen on In-Patient Settings) project aimed to develop and evaluate a stepped psychological intervention model to increase access to psychological therapy on acute mental health wards and consequently reduce serious incidents, improve patient well-being and functioning, and reduce staff burnout [7]. Thirty-four wards across England, Wales, and Northern Ireland participated in a cluster randomised control trial (RCT) to evaluate the effectiveness of the intervention. A process evaluation, embedded within the trial, aimed to provide additional insights into staff and patient perspectives on the impact of the intervention and contextual factors that impeded or enhanced intervention delivery [8].

The process evaluation comprised three strands: Semi-structured interviews with intervention users and providers, ethnographic observations on intervention wards [9], and fidelity assessments of intervention delivery [10]. This study presents findings from qualitative interviews with staff and patients and aimed to explore their views of the intervention and associated outcomes. The study also aimed to highlight any barriers and enablers to the implementation of, and staff and patient engagement with, the intervention. Throughout this report we refer to the recipients of care as patients as opposed to service users or consumers. This is the term we used throughout the TULIPS programme of research as selected by our patient advisory group following much discussion.

## Methods

### Design

Semi-structured interviews with staff and patients were used to create narratives of their experiences. These narratives were constructed jointly by interviewees and the interviewer but remained centred in the experience of participants [11]. Interviews were led by topic guides focused on participants’ views on the impact of the intervention and barriers and facilitators to delivery and uptake. Topic guides were informed by findings from earlier interviews exploring how to increase access to psychological therapy on acute wards [6] and were piloted with stakeholders (i.e. an individual with experience as an inpatient, a registered nurse from inpatient services and a clinical psychologist).

### Setting

Participants were recruited from acute mental health wards randomised to the intervention arm of theTULIPS trial. Acute mental health wards provide mental health treatment to individuals experiencing episodes of acute mental distress and posing risks to themselves or others. The average length of stay in the UK was 40 days in 2021/22 [3], although the range is likely wide. All intervention wards in the trial had between 13 and 23 beds. The main staff group on the wards were nurses or nursing assistants but wards also typically had sessional input from psychiatrists and occupational therapists.

### The intervention

The TULIPS intervention involved a stepped model of care with three levels [7]. Step one was a psychological formulation developed by a psychologist together with the ward team. In step two, nursing staff were trained and supervised by psychologists to deliver brief guided interventions for patients available in groups or on a one-to-one basis. At step three, patients could access up to 16 sessions of psychological therapy with psychologists. At least half a day’s training on mental health and psychological formulation was offered to all ward staff. An additional half a day’s training on specific problem areas likely to present on wards (e.g. psychosis, self-harm, low mood etc.) were offered to registered mental health nurses or other ward staff nominated by ward managers.

### Recruitment and procedure

Inclusion criteria for staff and patients were: (a) present on intervention wards during the study period; (b) informed consent to participate in the study including the audio recording of the interview. An additional inclusion criterion for patients was capacity to provide informed consent and sufficient levels of concentration to partake in an interview, as determined by the care team in conjunction with the researchers.

Participants were recruited until data saturation was reached. We used two different recruitment routes. Either intervention providers asked potential participants for their consent to be contacted by a researcher, or researchers approached participants directly when they were present on intervention wards conducting ethnographic observations. Participants were purposively sampled on the basis of direct exposure to the intervention. Efforts were made to recruit individuals from diverse backgrounds and with a variety of job roles. Risk and capacity assessments for patient participants were completed with a named nurse, responsible clinician, or member of patients’ community mental health team (when interviews took place following discharge). All potential participants were provided with information on the interview process and goals of the research via participant information sheets, reminded of the voluntary nature of the study and their right to withdraw, and were provided the opportunity to ask questions before informed consent was sought. If potential participants wished to consider their participation researchers arranged a time to get back in contact with them. Interviews were conducted privately and in person (at NHS sites or patient participant’s homes) or remotely (on the telephone or using Microsoft Teams) depending on participant choice and risk information. Demographic questionnaires were completed by participants prior to interview. If participants became distressed researchers would stop the interview and ask the participant if they would like to continue. Any concerns were handed over to the nurse in charge or community mental health team.

### Participants

#### Informed consent

was obtained from 65 participants (33 Patients; 32 Staff). Two patient participants withdrew consent prior to interview due to deteriorations in their mental health. One interview with a patient participant was excluded from analysis due to a poor-quality recording. Interviews with 30 patients and 32 staff members were included in analysis. Interviews ranged from 19 to 123 min in length (M = 53 min). Some participants were interviewed on more than one occasion to capture differences in their experience over time. See Table 1 for sample characteristics.

**Table 1:** Sample characteristics

		*n*	%
Patient Demographics			
Gender	Male Female	17 13	43 57
Ethnicity	White British White Irish Black African Mixed White and Black Caribbean Mixed White and Black African Black Caribbean and African Black Other	20 2 2 2 2 1 1	65 6 6 6 6 3 3
Marital Status	Single Married Divorced Separated Widowed Civil Partnership Common Law	21 3 2 1 1 1 2	68 10 6 3 3 3 6
Primary Diagnosis	Personality Disorder Schizophrenia Schizoaffective or psychosis Bipolar Anxiety Depression Obsessive Compulsive Disorder Aspergers No Formal Diagnosis	11 9 4 3 1 1 1 1 1	37 30 13 10 3 3 3 3 3
Additional Diagnoses	Anxiety Depression Personality Disorder Other None	12 12 6 2 9	39 39 19 7 29
Received therapy previously?	Yes No	23 8	74 26
Therapy received (*N* = 23)	Cognitive Behavioural Therapy Talking Therapy Mindfulness Dialectical Behavioural Therapy Cognitive Analytical Therapy Counselling Formulation Acceptance Commitment Therapy Other	12 13 6 2 1 1 1 1 2	44 41 19 6 3 3 3 3 6
Setting received in (*N* = 23)	Outpatient Inpatient Inpatient and Outpatient Not given	11 4 7 1	49 17 30 4
Staff Demographics			
Gender	Male Female	7 25	22 78
Ethnicity	White British Black African South Asian Mixed White and Black Caribbean	25 3 2 1	78 9 6 3
Job Role	Clinical Psychologist Healthcare Assistant Nurse Ward Manager Occupational Therapist Psychiatrist Activities Co-ordinator Deputy Ward Manager Assistant Psychologist	6 6 5 5 3 3 2 1 1	19 19 16 16 9 9 6 3 3
Band*	Band 2 Band 3 Band 4 Band 5 Band 6 Band 7 Band 8a Band 8b > Band 8c Not applicable Not Given	3 3 2 3 4 6 3 1 2 3 2	9 9 6 9 13 19 9 3 6 9 6
Years Experience in Mental Health**	< 1 year 1–2 years 2–5 years 5–10 years > 10 years	11 3 5 8 12	34 9 16 25 38
Years Experience in Acute**	< 1 year 1–2 years 2–5 years 5–10 years > 10 years	4 1 6 9 12	13 3 19 28 38
Training in psychological interventions	Yes No	21 11	66 34
Therapies Trained in (*N* = 21)	Cognitive Behavioural Therapy Mindfulness Other Dialectical Behavioural Therapy Cognitive Analytical Therapy Acceptance Commitment Therapy Compassion Focused Schema Therapy Meta Cognitive Therapy Eye Movement Desensitization and Reprocessing Talking Therapy Counselling Formulation	11 11 7 5 5 5 4 3 2 2 2 1 1	52 52 33 24 24 24 19 14 10 10 10 3 3
Delivery Setting (*N* = 21)	Inpatient Outpatient Inpatient and Outpatient Not Given	8 3 5 5	38 10 24 24

Note. Other therapies included art and music therapy

*All non-medical jobs in the UK National Health Services are allocated a Band which is based on skill set required to carry out the job and is used to determine the salary. There are 9 bands in total with 9 representing the highest band in terms of skill and pay. Bands are also split into sub-bands (e.g. 8a, 8b, 8c etc.)

**Categorial data so no means, standard deviations and ranges provided

### Analysis

All interviews were audio recorded, transcribed verbatim and analysed inductively using reflexive thematic analysis [12, 13]. The coding was carried out with Nvivo 12 software. The following stages of analysis were completed separately for the staff and patient interviews. Firstly, a small team of three researchers coded the same transcript. A consensus meeting was then held to discuss differences in interpretation, develop a shared meaning and create a code book. Transcripts were then randomly assigned to coders and the codebook applied. All data felt to be incongruent with the codebook was labelled with ‘other’ and discussed during regular coding meetings leading to adaptations of the codebook. These meetings also facilitated discussions of emerging themes, reflections and the renaming and reorganising of codes.

In addition to the initial consensus checking, 30% of transcripts were coded by at least two researchers. We initially aimed for 25% of the transcripts to be double coded but due to the addition of new coders to this team this rose to 30%. Double coding is a common practice to consensus check and test the validity of codebooks used [14]. As researchers began analysis, we ensured there was overlap with multiple researchers coding the same transcript. As the codebook become more refined and researchers more confident and familiar with the codebook this tapered off. At points throughout analysis new coders who had not been involved in the original consensus checking joined the team, therefore we repeated this process to ensure codes had a shared meaning among the whole team. Having multiple coders work on the same transcript enabled us to bring in different perspectives based on the varying experience and backgrounds of the team. It also enabled us to check the validity of the codebook by directly comparing if two different researchers used the same code for a section of the transcripts.

Where there were discrepancies in coding these were overcome through discussion, review of reflexive diaries and the raw data. Once all transcripts from that participant group were coded, theme development meetings were held where codes were organised into domain summaries. During these meetings, emergent themes discussed throughout the analysis were expanded on and additional themes were identified through reviewing the final codebook and raw data. This process was iterative and reflexive, informed by researchers’ knowledge of the data, experience conducting interviews and awareness of the wider study [13]. Initial themes (eight staff themes and six patient themes) were written up by author1 and refined based on feedback from the coding team.

Initial themes where then presented to three members of the TULIPS patient and carer group during validation workshops [15]. All members had a lived experienced of inpatient wards as a patient or carer. Quotes felt to be most representative of each theme were presented without interpretation and members were asked open questions to encourage discussion and gain their perceptions of the raw data. Interpretations were explored and detailed minutes taken. These interpretations were then compared with the research teams and helped to further refine or validate themes. Participants themselves did not provide feedback on transcripts or themes.

Once the aforementioned steps were completed for both staff and patient data, associated themes were organised into the domains of the Consolidated Framework for Implementation Science Research (CFIR) [16]. When themes from staff and patient interviews were associated with the same domain these were extracted, compared and, where appropriate, interwoven to create rich themes accounting for both staff and patient perspectives. We initially aimed to conduct a framework analysis informed by CFIR but after indexing we found our data transcended the domains. Instead, our analysis remained thematic with CFIR being utilised only to organise and combine datasets, but not influencing interpretations. Analysis continued throughout the writing process with feedback from the wider study team contributing to refinements in themes.

### Reflexivity

All authors involved in the collection and analysis of data were female and employed researchers on the TULIPS project. Interviews were predominantly conducted by experienced qualitative researchers: author 1 and 7. Author 1 also led data analysis. All but one author involved in data collection and analysis had experience working in inpatient mental health settings. Researchers therefore already held understandings of ward environments, processes and the local language used, providing context to interviewees’ experiences. This background influenced interpretations with researchers being more likely to focus on salient features of their own ward experiences, such as wards lacking resources and conflict within staff-patient relationships. The interviewers had spent time on the wards as participant observers prior to the interviews but were not well known to the interviewees.

Reflexive journals were reflected on during analysis meetings enabling critical reflections, transparency, and credibility [17]. Researchers discussed excerpts of their journals during analysis meetings, with particular emphasis on how researchers’ professional and personal experiences led to certain transcripts or perspectives being more poignant. For example, there was discussion about psychologists’ characteristics and whether these were influenced by personality or professional training, as these were initially distinct codes. After review of the raw data and critical reflection on the coders’ personal beliefs, it was agreed to code data pertaining to characteristics as a therapeutic skill, but it was noted that patients often perceived therapeutic skills as being part of the psychologist’s personality.

## Results

There were four themes in total. The first theme is entitled ‘respected but unique part of the team’ and was developed from both staff and patient data. The second theme is entitled, ‘facilitating understanding’ and was also developed from both staff and patient data. The third theme is entitled, ‘beliefs about job role’ and was developed from staff data only. The fourth theme, entitled ‘putting psychologists on a pedestal’ was developed from both staff and patient data. Perceived enablers to implementation and engagement, and associated outcomes are discussed across these themes and summarised in Fig. 1 (see supplementary material).

### Respected but unique “*part of the team*”

This theme describes the role that psychologists had within the team from the perspectives of staff, patients and the psychologists’ themselves, and provides explanations of what might have influenced these perceptions.

Psychologists were regularly visible on wards engaging with patients and spending time in the nursing office which afforded them status as a member of the ward team. Psychologists regular physical presence coupled with their flexibility and persistence (adding meetings to diaries, reminding staff and re-booking when cancelled) had multiple benefits for ward staff including accessible consultation about how to respond to patients’ behaviours and opportunities for emotional support during an emotionally draining shift. The changeable nature of ward environments meant staff could not always attend scheduled meetings with the psychologist, but psychologists physical presence enabled staff to access support in vivo.

*Things run over but if you’ve got a psychologist there and they’re around and they’re visible you can just say oh have you got two minutes to talk about this.*** Psychiatrist A**.

Psychologists presence was also essential in providing opportunities to develop relationships with staff further enhancing integration within the team. Informal social interactions outside of meetings were especially important for this rapport building. Being around and willing to help with tasks outside the remit of the psychologist’s role afforded respect from nursing staff and led to beliefs that psychologists recognised the difficulties of the nurse’s job. Psychologists also recognised the importance of these relationships for the implementation of the intervention noting they needed to be an insider to facilitate change:

*In order to influence culture you need to not be a threat and you need to have relationships with people.*** Psychologist A**.

Developing relationships with psychiatrists also aided psychologists integration within the wider multidisciplinary team and led psychiatrists to encourage patients to engage in psychological assessment or intervention. This endorsement from psychiatrists who were senior team members indicated that the intervention was credible and should be prioritised.

*It made a huge difference if you’ve got a psychiatrist endorsing it*,* who is considered someone who is considered*,* the lead clinician on the ward. I suppose that gives you a bit of kudos by association.*** Psychologist A**.

The psychologists effort to embed themselves within staff teams acted as a mechanism for staff and patient engagement with the intervention. Yet, maintaining some independence from the team by virtue of being from a different professional group was also beneficial. On wards with significant staff conflict, or hierarchal cultures, members of the nursing team felt able to confide in psychologists about their concerns due to their perceived understanding of the ward environment and some degree of impartiality.

*They are a team member but you’re not directly a team member*,* so it would be nice sometimes just to*,* offload to someone. *** Nursing Assistant A**.

Psychologists perceived impartiality and independence from the nursing team coupled with their provision of a dedicated safe space also fostered patient engagement. These patients described previous negative experiences of care on acute wards leading to a mistrust of nursing staff on the team.

*We were in a safe space and that’s what the psychologist has to create. A place*,* you know*,* someone to talk to that is third-party neutral. That’s important.*** Patient A**.

### Facilitating Understanding

This theme describes how both staff and patients perceived that the psychologist was able to improve their understanding of the patients needs and behaviours. In the case of patients, this theme represented improved self-understanding through therapy and in the case of staff this theme involved psychologists helping staff to formulate the reasons for patients behaviours.

Patients described how one-to-one sessions with psychologists facilitated greater understanding of how life experiences related to current emotions and behavioural patterns.

*I understood why I feel the way I do and act the way I do*,* and when I was able to sort of express myself*,* then I could understand myself more.*** Patient D**.

This experience differed from treatment prior to the intervention where patients described a process of “*telling their story over and over to different people*” [Patient B] without opportunities to understand the reasons for their distress or help in developing coping strategies.

Some patients were anxious about the idea of making themselves vulnerable within the context of therapy, particularly for those reporting prior negative experiences of counselling or therapy. However, through showing understanding, psychologists were able to put these anxieties to rest.

*You feel like [the psychologist] is taking an interest in your particular situation and then*,* you can then work with them better. You feel they are trying to understand me*,* not just a condition.*** Patient J**.

Staff also perceived that the intervention improved patients self-understanding and described associated reductions in self-harm, aggression, and negative affect.

*What I’ve noticed is that patients feel much safer when they have interactions with the psychologist and the number of incidents decrease*,* and they feel like there’s somebody really listening and understanding.*** Psychiatrist B**.

As well as enabling patients self-understanding, psychologists facilitated staff knowledge of patient needs through different forums. Staff reported that attending psychologist-led formulation sessions increased their understanding of the meaning, and motivations behind behaviours that typically attracted staff criticism (e.g. aggression, self-harm).

*I have better understanding of why*,* if someone’s behaving- if someone displays challenging behaviour should I say it helps me to have a better understanding of where they’re coming from.*** Occupational Therapist B**.

Patients also welcomed the psychologist sharing aspects of their formulation with staff to improve staff understanding of support needs.

*[The psychologist] can go to members of staff and say*,* he struggles with this*,* struggles with that*,* so the members of staff on the ward are getting a better insight in how you’re dealing with*,* the certain situations*,* which is a massive thing cos some of staff won’t even know.*** Patient L**.

These examples of knowledge sharing were described in terms of psychologists acting as a *bridge* between patients and staff, suggesting staff and patients previously felt disconnected despite being physically present on the wards together much of the time. Understanding the functions of patient behaviour also helped to enable compassion and empathy, which staff associated with an increased feeling of reward from their work.

*If we’d had a service user that is continually displaying aggressive behaviour*,* some staff find it really difficult to deal with*,* and they start almost feeling burnt out*,* and then I think the formulations and the one-to-one sessions have really helped staff take a step back and think about actually this is what’s triggered this behaviour It’s kind of helped people sort of be more empathetic*.** Ward Manager A**.

Some patients perceived that staff had become more compassionate and “*less judgemental*” [Patient C] as a result of the intervention. Others believed that care had not improved as levels of compassion were dependent on individual differences among staff suggesting little optimism about future changes in staff behaviour.

*It was only like a couple of [staff] members that that would do their utmost to cheer you up and make sure you’re okay. But a vast majority of them would just like*,* you might as well not be there at all.*** Patient K**.

Formulations were also shared with community or home treatment teams. As a result, some patients perceived that step-down services were better equipped to continue supporting recovery.

*My care coordinator*,* she had a session with [psychologist]*,* and they spoke about the formulation that we’d done so I don’t have to repeat myself again and sort of talk about a lot of things.*** Patient D**.

However, some patients described feeling “*disappoint[ed]”* [Patient M] at not being able to continue engaging in the intervention after discharge, preventing them from maintaining the level of functioning developed. Although patients still perceived the ward-based intervention to be beneficial, some felt it should extend into the community.

*I would have liked to continue it to be honest especially as an outpatient because whilst sessions with her with good*,* it was just*,* it would have been nice to have it followed up.*** Patient C**.

### Beliefs around job role

This theme describes how beliefs about the function of acute mental health care and the nurse’s role within it hindered nurses involvement in delivering therapy and engaging with sessions delivered by the psychologists. We also describe what factors helped to improve engagement and counter barriers associated with these beliefs.

Staff described how acute wards served to stabilise patients and reduce risk with an aim to discharge them as soon as possible. This perception of ward function, combined with staff beliefs that psychological therapy is lengthy, complex, and unsuitable for patients with schizophrenia, led some staff to believe that the intervention was at odds with ward aims.

*How do you embed the psychology into the purpose of the ward? ‘Cos if that purpose is to be a really acute short stay ward that rapidly turns around people*,* has bed availability and all those kind of things*,* that doesn’t tend to lend to lengthy*,* more consistent interventions. *** Ward Manager B**.

Narratives around the function of acute wards fed into beliefs that nursing staff’s primary role did not include providing psychological therapy. Staff typically depicted their key responsibilities to be medication administration, risk management and completing paperwork. These beliefs likely limited staff engagement in delivering nurse-led interventions as these were seen as “*low priority*” [Occupational Therapist C]. Although staff busyness with other duties was a significant barrier to intervention implementation, some staff did successfully deliver interventions. These individuals prioritised intervention delivery over other tasks.

Some staff described how formulation sessions provided a safe and non-judgemental space to challenge rhetoric around the limits of their job role. For these individuals engaging with psychologists led to perceptions that their responsibilities should include providing therapeutic support to patients. For some staff this change in belief encouraged engagement in intervention delivery.

*I’m not just there to unlock doors and to give people food*,* it (formulation sessions) reminds me that*,* in our everyday interactions with patients on the ward there’s a therapeutic reason for nursing assistants to be here. *** Nursing Assistant C**.

However, this prioritisation of the intervention was primarily enabled by ward managers buy-in which was felt to be *instrumental* in enabling staff to prioritise engaging in interventions over other activities.

*The way that it was talked about within the team was maybe like this is a nice optional extra*,* but it’s not essential*,* whereas on the other ward the ward manager really kind of prioritised staff being able to spend time with me.*** Psychologist B**.

Unlike other members of the multi-disciplinary team, nursing staff lacked control over their time, meaning that without ward manager support they were unable to dedicate time to the intervention.

*(Nurses) turn up on shift and you’re told what you’re doing for each hour and psychological work isn’t part of that scheduling.*** Psychologist C**.

As a result, in the absence of ward manager support, level 2 interventions were most successfully led by staff who were in control of their diaries and not expected to respond to incidents, such as assistant psychologists and occupational therapy staff (including recovery workers and activity co-ordinators).

### Putting psychologists on a pedestal

This theme describes how patients perceived psychologists compared with nursing staff and what factors influenced these perceptions. It also describes psychologist’s perception that staff turned to psychological therapy as a solution for patients they found difficult as opposed to trying to problem solve together with the whole team.

Psychologists flexible schedule enabled protected time and space to develop therapeutic alliance with patients. This ability to give uninterrupted, quality time was highly valued, and enabled patient engagement. In contrast, nursing staff lacked opportunities to devote time to patients and had other priorities as a part of their role which acted as an obstacle to patients seeking support.

*If something happens on the ward*,* and that nurse has to go. You might be allocated a time*,* but that necessarily might not happen. You can never get through a full meeting*,* without something happening on the ward*,* and then you’re more reluctant to speak to them.*** Patient M**.

Perceptions that psychologists held a higher level of therapeutic training and expertise also motivated patients to engage with psychologist-led sessions.

*It’s the knowledge and the understanding of one’s emotions or what they’re going through. I’m not saying the nurses aren’t understanding*,* but when someone’s trained in that speciality of being a psychologist*,* they just have more of an understanding.*** Patient M**.

Conversely, beliefs nursing staff were “*not very confident”* [Patient N] in delivering psychological interventions reduced patients’ motivation to engage in nurse-led interventions. Nursing staff themselves also reported a lack of confidence, due partly to few opportunities to build experience and skill in intervention delivery.

*That is one of the difficulties with all the interventions we do is that there is always the sense that you’re learning as you’re going*,* so-so the first few that you do*,* it’s almost like you’re not 100% clear what you’re trying to work through with someone. Sometimes that can be off-putting.*** Nurse A**.

Psychologists felt ward environments were not conducive of staff building confidence and learning new skills, such as those required to deliver interventions.

*I think you just stay in your comfort zone*,* when you’re just feeling like you’re spinning plates*,* or you feel a bit stressed out in a job*,* so I think*,* staff confidence has impacted on their delivery of the interventions.*** Psychologist A**.

Perceptions that support provided by psychologists was superior meant patients did not always see the benefit of engaging in nurse-led interventions. This perception contributed to a dichotomy in patient’s perceptions of staff, with psychologist who had the luxury of managing their own diary and more training in therapies being put on a pedestal whilst nursing staff who had multiple competing demands and skill sets were described more critically.

Psychologists also perceived that ward staff had high expectations of their role. They described how staff would refer patients for therapy who were difficult to manage rather than seeking advice and engaging in intervention delivery to adapt their own practice. In this way, staff’s perception that psychologists filled a gap in treatment was in some ways at odds with one of the main aims of the intervention: to upskill staff to engage more therapeutically with patients.

*I still think that’s why sometimes people will say oh will you see this person? Often the medic will sort of tickety tick that box that*,* they’re seeing our psychologist that’s great then they feel okay.*** Supervisor A**.

## Discussion

This study aimed to explore staff and patients views of the TULIPS intervention and associated outcomes. The study also aimed to highlight any barriers and enablers to implementation of and staff and patient engagement with the intervention. Both staff and patients had positive perceptions of the stepped model of psychological intervention in terms of its perceived impact on patients’ understanding of their emotional distress and also staff members’ understanding of patient needs. This improved understanding was facilitated by psychologists’ availability and frequent physical presence on the wards, as well as their focus on relationship building with both staff and patients. However, the perception that psychological therapy was not part of the nurse’s role and lack of support from managers to dedicate time to therapy meant that the nurse-led interventions were not delivered frequently or with sufficient skill and confidence.

The psychologists frequent physical presence on the wards enabled psychologist’s to be flexible and adaptative in response to the ward environment. This flexibility and adaptation are well-established factors in enabling the successful delivery of complex interventions [16] but may be particularly important on acute mental health wards due rapid escalations in patient distress and risk and the consequent focus on crisis management [18]. The focus on relationship building is another well-known factor in the successful delivery of complex interventions and ward-based interventions in particular [19]. However, previous research has found that psychologists can be seen unwelcome ‘experts’ within teams [20, 21] suggesting that psychologists may not routinely invest time in this important foundation for delivery psychological consultation or therapies.

The role of psychological formulation in facilitating understanding of patients needs has previously been shown in studies focused on both patient perspectives of formulation [22] and staff perspectives of team formulation [22, 23]. The fact that patients reported a contrast between their experiences of therapy with the psychologists and their previous experiences of assessments within ward environments is reflective of the limited availability of psychological therapies on acute wards [24]. Previous studies have suggested that psychological therapies might not be compatible with acute ward culture [5, 6] and some staff we interviewed echoed these concerns. However, our findings coupled with findings from other research [6] consistently highlights that patients value the opportunity of therapy as an inpatient and do not share staff concerns.

The finding that improved understanding of patient needs through team formulation improved staff empathy and compassion is also consistent with previous research exploring the effects of team formulation [22] and is one of the key rationales for the team formulation process [25]. Nonetheless, previous research has reported negative effects of team formulation including the perception that formulations devalue staff members’ current ways of working [26]. The risk of staff perceiving formulation in this way could have been mitigated in our study by the focus psychologists placed on relationship building with staff alongside the provision of formulation and other aspects of the stepped model of care. Although there is evidence that formulations can impact on staffs’ self-reported understanding and empathy, both our study and previous research [26] suggests some staff do not change their practice as a result of team formulation. Greater attention therefore needs to be paid to ensuring that the recommendations of formulation are fed into care planning. Team formulation meetings in our study and in previous research [22] were primarily attended by nursing staff, but involving psychiatrists more routinely in the process may help to ensure that the information generated feeds into all aspects of the person’s care.

The finding that some nurses viewed psychological therapies as outside of their role may reflect the broad nature of the nurse’s role [27]. Nurses are frontline staff and represent a large workforce within the health service. Many ward processes and procedures are therefore typically allocated to nurses meaning that they have many competing demands [28]. In the context of a risk averse environment where blame and accountability are high and nurses are tasked with fulfilling organisational restrictions, the focus of nurse’s time and attention easily gets drawn into processes that are mandatory and auditable and the documentation of these processes [29, 30]. Despite competing demands, our study and previous research suggests that nurses can ring fence time to deliver psychological therapies with managerial support [31, 32]. Engaging ward managers in the delivery of the stepped model of care is therefore vital from the outset. The CFIR framework for implementation highlights the important role of shared values, beliefs and norms in order to change organisational culture, with shared values playing a pivotal role guiding the behaviour of staff and managers [16]. Ensuring that managers and staff value the ethos and key components of the TULIPS intervention is therefore essential to ensuring wider adaptation. Witnessing the positive impact that psychological therapy can have on patient and ward outcomes can be a key factor in shifting ward staff values. It would also be important to share positive firsthand accounts around the benefits of psychological therapy within acute care settings from patient and staff perspectives through a variety of different forums.

The finding that inpatient mental health nurse’s lack confidence in delivery the intervention was not surprising given the limited opportunity that some staff had in delivery due to the custodial nature of their role. A total of five nurses (all trained and registered mental health nurses) and six nursing assistants/health care assistants were interviewed within this study. Notably, there was no distinction between the registered nurse and nursing assistant self-reported skill level and confidence in engaging in intervention delivery suggesting that nursing degrees did not equip staff to carry out psychological interventions. Time for psychologists to continue to support and supervise staff in delivering interventions post training is therefore an essential component of any intervention that aims to upskill nurses to deliver psychological interventions. The perceived dichotomy between psychologists and nursing staff, from patients perspective, was an unintended consequence of the intervention, but reflects the multiple demands on the nurse’s time which prevents one-to-one work with patients, the work that presumably motivated them to join the profession in the first place [33]. Supporting nurses to be able to develop skills and practice therapeutic work onwards is therefore essential in recruiting and retaining a motivated and satisfied workforce who are able to deliver the best care possible.

### Strengths and limitations

This study is a large qualitative study which captures a range of different perspectives on the implementation of a ward-based psychosocial intervention. It is strengthened by the existence of additional quantitative and qualitative (observational) data that will be synthesised to enable a broader understanding of the effects of the intervention and barriers and facilitators to delivery. Nonetheless there are several study limitations to note. The sample was limited in that patients from ethnic minority backgrounds are under-represented. Attempts were made to purposively recruit patients who declined to participate in the intervention, however, none consented to be interviewed. These individuals may have held distinct views on the intervention and trial psychologists, which we were unable to capture. Although members of the patient and carer group were involved in validating themes, conducting member checking with participants would have strengthen the methodology.

Although two of the paper authors are mental health nurses, there were also no nurses directly involved within the process of data collection and analysis who may have brought valuable perspectives to the interviews. Similarly, although our patient advisory group were involved in the analysis of the data, employing interviewers with lived experience of mental health inpatient care, may have improved the interview experience for patients and encouraged them to open up about negative perceptions of the intervention.

## Conclusions

From the perspectives of staff and patients, the TULIPS intervention positively influenced their ward experience leading to reports of improved self-understanding for patients and of greater understanding and compassion among staff. However, the uptake of nurse-led interventions was relatively poor, due to beliefs about the function of acute wards, the remit of the nurse’s role, limited support from ward managers and the consequent lack of nurse confidence in intervention delivery. Wider system level changes are needed for nurse-led psychological interventions to be feasible within inpatient settings. Such changes could include a greater focus on psychological interventions during nursing training and as part of continued professional development, the delivery of therapeutic interventions in job role descriptions and the inpatient leads ensuring that staff have protected time to facilitate these tasks.

### Relevance for clinical practice

A stepped model of care with formulations as a foundation is welcomed by patients and ward staff. Patients and staff also benefit from the presence of a ward-based psychologists who can provide regular opportunistic consultation to staff and therapy to patients. However, further work is needed to enable nurses to put psychological therapies into practice on the wards and develop skills in this area. This change will necessitate support from senior ward staff including ward managers but also changes to professional training, continued professional development and job descriptions. Such changes are important in terms of improving patients’ experience of the ward but also helping the recruitment and retention of nurses within inpatient settings.

## Electronic supplementary material

Below is the link to the electronic supplementary material.

Supplementary Material 1: Figure 1 Thematic Map

## Data Availability

Data is available from the corresponding author upon reasonable request.

## Abbreviations

TULIPS: Talk Understand and Listen in In-Patient Settings
RCT: Randomised Controlled Trial
NICE: The National Institute for Health and Care Excellence
UK: United Kingdom
NHS: National Health Service
CFIR: Consolidated Framework for Implementation Research

## References

[1] National Institute for Health and Care Excellence. Schizophrenia: Core Interventions in the Treatment and Management of Schizophrenia in Primary and Secondary Care (Update). 2014. https://www.ncbi.nlm.nih.gov/books/NBK11681/. Accessed 25 March 2024.

[2] Penfold N, Nugent A, Clarke H, Colwill A. Standards for acute inpatient services for Working-Age adults. London: Royal College of Psychiatrist; 2019.

[3] National Health Service. Acute inpatient mental health care for adults and older adults, NHS England. 2023. https://www.england.nhs.uk/long-read/acute-inpatient-mental-health-care-for-adults-and-older-adults/#:~:text=Older%20adults%20and%20people%20with%20dementia%26text=For%20example%2C%20in%202021%2F22,in%20general%20adult%20acute%20services. Accessed: 25 March 2024.

[4] Burgess-Barr S, Nicholas E, Venus B, Singh N, Nethercott A, Taylor G, Jacobsen P. International rates of receipt of psychological therapy for psychosis and schizophrenia: systematic review and meta-analysis. Int J Mental Health Syst. 2023;17(1):8. 10.1186/s13033-023-00576-9 PMC10064673 37004066

[5] Evlat G, Wood L, Glover N. A systematic review of the implementation of psychological therapies in acute mental health inpatient settings. Clin Psychol Psychother. 2021;28(6):1574–86. 33870590 10.1002/cpp.2600

[6] Berry K, Raphael J, Haddock G, Bucci S, Price O, Lovell K, Drake RJ, Clayton J, Penn G, Edge D. Exploring how to improve access to psychological therapies on acute mental health wards from the perspectives of patients, families and mental health staff: qualitative study. BJPsych Open. 2022;8(4):e112. 35698827 10.1192/bjo.2022.513 PMC9230441

[7] Berry K, Raphael J, Wilson H, Bucci S, Drake RJ, Edge D, Emsley R, Gilworth G, Lovell K, Odebiyi B, Price O, Sutton M, Winter R, Haddock G. A cluster randomised controlled trial of a ward-based intervention to improve access to psychologically-informed care and psychological therapy for mental health in-patients. BMC Psychiatry. 2022;22(1):82. 10.1186/s12888-022-03696-7. 35114980 10.1186/s12888-022-03696-7 PMC8815159

[8] Moore GF, Audrey S, Barker M, Bond L, Bonell C, Hardeman W, Moore L, O’Cathain A, Tinati T, Wight D, Baird J. Process evaluation of complex interventions: medical research Council guidance. BMJ. 2015;350. 10.1136/bmj.h1258 PMC4366184 25791983

[9] Berry K, Johnston I, Wilson P, Haddock G, Bucci S, Lovell K, Price O, Beinaraviciute A, Gilworth G, Kaur S, Morley H, Pennington-Smith G, Raphael J, Samji M, Drake RJ. Edge, D. Barriers to the implementation of psychosocial interventions on acute mental health wards: An ethnographic observational study. *Frontiers in Psychiatry*. In press. 10.3389/fpsyt.2025.1501945 PMC11920708 40109435

[10] Berry K, Handerer F, Bucci S, Penn G, Morley H, Raphael J, Lovell K, Price O, Edge D, Drake RJ, Haddock G. Ensuring that psychological interventions are delivered as intended on mental health inpatient wards. Br J Clin Psychol. 2024 Oct;28. 10.1111/bjc.12510. Epub ahead of print. 10.1111/bjc.12510 PMC12057325 39467006

[11] Gubrium JF. Active interviewing. In: Holstein JA, editor. Postmodern interviewing. London: Sage; 2003. pp. 67–80.

[12] Braun V, Clarke V. Using thematic analysis in psychology. Qualitative Res Psychol. 2006;3(2):77–101.

[13] Braun V, Clarke V. Reflecting on reflexive thematic analysis. Qualitative Res Sport Exerc Health. 2019;11(4):589–97.

[14] Raskind IG, Shelton RC, Comeau DL, Cooper HLF, Griffith DM, Kegler MC. A review of qualitative data analysis practices in health education and health behavior research. Health Educ Behav. 2019;46(1):32–9. 30227078 10.1177/1090198118795019 PMC6386595

[15] Sprange K, Beresford-Dent J, Mountain G, Thomas B, Wright J, Mason C, Cooper CL. Journeying through dementia randomised controlled trial of a psychosocial intervention for people living with early dementia: embedded qualitative study with participants, carers and interventionists. Clin Interventions Aging 2021 Feb 4:231–44. 10.2147/CIA.S293921 PMC7872215 33574660

[16] Damschroder LJ, Reardon CM, Widerquist MA, Lowery J. The updated consolidated framework for implementation research based on user feedback. Implement Sci. 2022;17(1):75. 36309746 10.1186/s13012-022-01245-0 PMC9617234

[17] Ortlipp M. Keeping and using reflective journals in the qualitative research process. Qualitative Rep. 2008;13(4):695–705.

[18] Wood L, Williams C, Billings J, Johnson S. Psychologists’ perspectives on the implementation of psychological therapy for psychosis in the acute psychiatric inpatient setting. Qual Health Res. 2019;29(14):2048–56. 31014190 10.1177/1049732319843499

[19] Raphael J, Price O, Hartley S, Haddock G, Bucci S, Berry K. Overcoming barriers to implementing ward-based psychosocial interventions in acute inpatient mental health settings: A meta-synthesis. Int J Nurs Stud. 2021;115:103870. 33486388 10.1016/j.ijnurstu.2021.103870

[20] Christofides S, Johnstone L, Musa M. Chipping in’: clinical psychologists’ descriptions of their use of formulation in multidisciplinary team working. Psychol Psychotherapy: Theory Res Pract. 2012;85(4):424–35. 10.1111/j.2044-8341.2011.02041.x 23080531

[21] McKenna M, Brown LJ, Berry K. Formulation-led care in care homes: staff perspectives on this psychological approach to managing behaviour in dementia care. Int J Older People Nurs. 2022;17(5):e12465. 35403365 10.1111/opn.12465 PMC9541292

[22] Berry K, Haddock G, Kellett S, Awenat Y, Szpak K, Barrowclough C. Understanding outcomes in a randomized controlled trial of a ward-based intervention on psychiatric inpatient wards: A qualitative analysis of staff and patient experiences. J Clin Psychol. 2017;73(10):1211–25. 28026872 10.1002/jclp.22434

[23] Bealey R, Bowden G, Fisher P. A systematic review of team formulations in multidisciplinary teams: staff views and opinions. J Humanistic Psychol. 2021 Sep;7:00221678211043002.

[24] Staniszewska S, Mockford C, Chadburn G, Fenton SJ, Bhui K, Larkin M, Newton E, Crepaz-Keay D, Griffiths F, Weich S. Experiences of in-patient mental health services: systematic review. Br J Psychiatry. 2019;214(6):329–38. 30894243 10.1192/bjp.2019.22

[25] Association of Clinical Psychologists. Team formulation: Key considerations in mental health services. 2022. https://acpuk.org.uk/wp-content/uploads/2022/07/ACP-UK-Team-Formulation-Guidance-v1.pdf. Accessed: 25 March 2024.

[26] Geach N, Moghaddam NG, De Boos D. A systematic review of team formulation in clinical psychology practice: definition, implementation, and outcomes. Psychol Psychotherapy: Theory Res Pract. 2018;91(2):186–215. 10.1111/papt.12155 28972700

[27] Totman J, Hundt GL, Wearn E, Paul M, Johnson S. Factors affecting staff morale on inpatient mental health wards in England: a qualitative investigation. BMC Psychiatry. 2011;11:1–0. 21510852 10.1186/1471-244X-11-68 PMC3103421

[28] Wyder M, Ehrlich C, Crompton D, McArthur L, Delaforce C, Dziopa F, Ramon S, Powell E. Nurses experiences of delivering care in acute inpatient mental health settings: A narrative synthesis of the literature. Int J Ment Health Nurs. 2017;26(6):527–40. 28295948 10.1111/inm.12315

[29] Ball H, Yung A, Bucci S. Staff perspectives on the barriers and facilitators to exercise implementation in inpatient mental health services: A qualitative study. Ment Health Phys Act. 2022;22:100452.

[30] Bowers L, Simpson A, Alexander J, Hackney D, Nijman H, Grange A, Warren J. The nature and purpose of acute psychiatric wards: the Tompkins acute ward study. J Mental Health. 2005;14(6):625–35.

[31] James K, Quirk A, Patterson S, Brennan G, Stewart D. Quality of intervention delivery in a cluster randomised controlled trial: a qualitative observational study with lessons for fidelity. Trials. 2017;18:1–10. 29149915 10.1186/s13063-017-2189-8 PMC5693543

[32] Radcliffe JJ, Adeshokan EO, Thompson PC, Bakowski AJ. Meeting the needs of families and carers on acute psychiatric wards: a nurse-led service. J Psychiatr Ment Health Nurs. 2012;19(8):751–7. 22762305 10.1111/j.1365-2850.2012.01932.x

[33] Edwards K. What prevents one to one care? Nurs Times. 2011;107(1):25–7. 21313940

