# Supplementary figures and images for: Increasing access to psychological therapy on acute mental health wards: staff and patient experiences of a stepped psychological intervention
Source: BMC Psychiatry. 2025 Mar 28;25:300. doi: 10.1186/s12888-025-06721-7 (PMC11954329; doi:10.1186/s12888-025-06721-7)

**Figure 1 – supplementary material**

Thematic Map


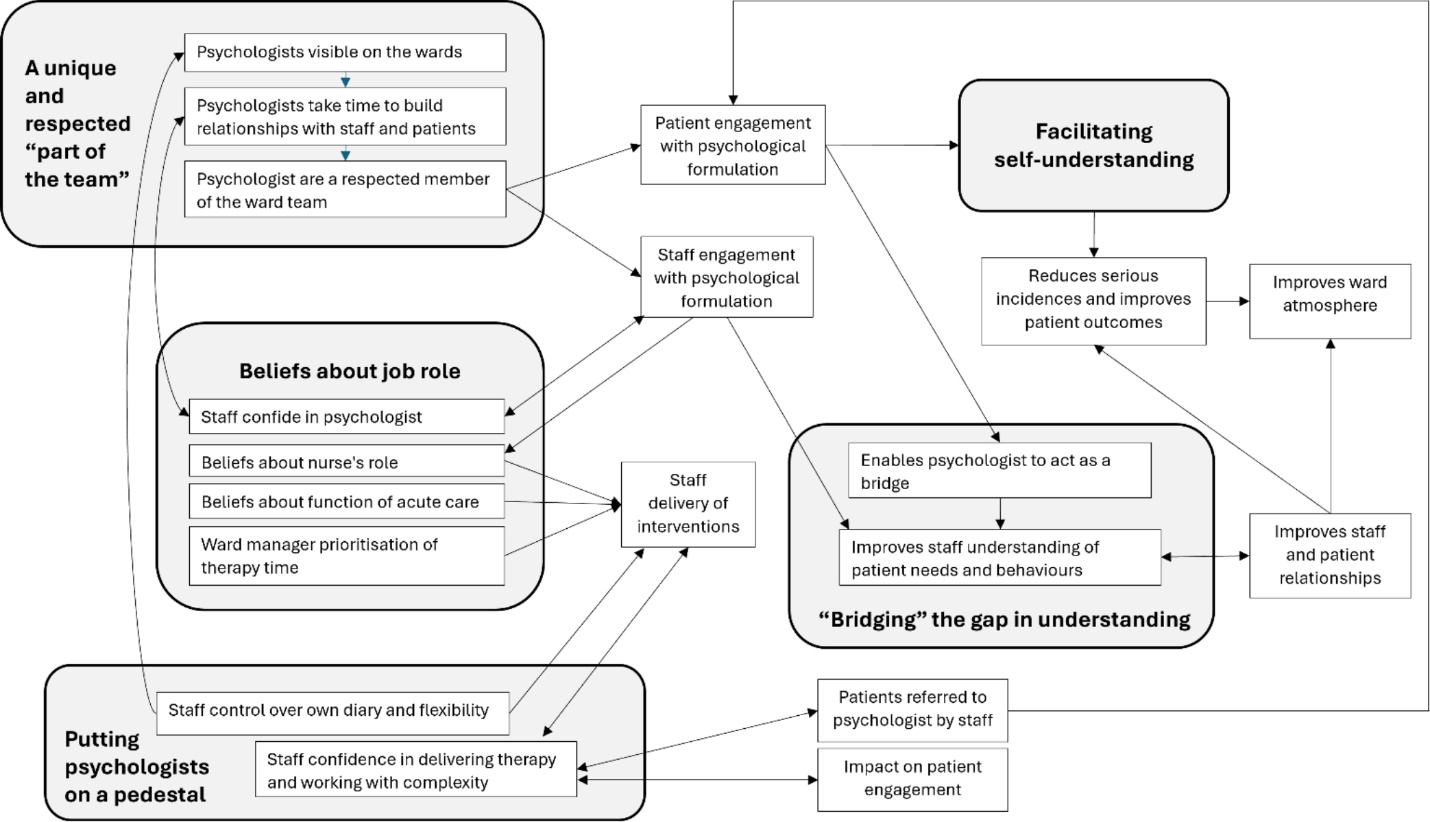

Supplement: Supplementary file 1 — Supplementary Material 1: Figure 1 Thematic Map [file 12888_2025_6721_MOESM1_ESM.docx]
